# Representations of mental health, madness, mental illness and depression in the general population of French Guiana: Similarities and differences between sociodemographic subgroups

**DOI:** 10.1371/journal.pmen.0000655

**Published:** 2026-08-13

**Authors:** Mathieu Nacher, Astrid Van Melle, Estelle Thomas, Johanna Pavie, Blandine Solignat, Imane Benradia, Deborah Sebbane, Jean-Luc Roelandt, Caroline Janvier, François Lair, Vincent Bobillier

**Affiliations:** 1 Centre d’investigation Clinique Institut National de la Santé et de la Recherche Médicale (CIC Inserm) 1424, Centre Hospitalier Universitaire (CHU) de French Guiana, Cayenne, French Guiana; 2 UA17 Inserm Santé des Populations en Amazonie, Université de French Guiana, Cayenne, French Guiana; 3 Plateforme Rétablissement, Groupe SOS, Cayenne, French Guiana; 4 CHU French Guiana, Cayenne, French Guiana; 5 Centre collaborateur de l’Organisation Mondiale de la santé (CCOMS) Pour la Recherche et la Formation en Santé Mentale – OMS. Service de l’établissement Publique de santé Mentale (EPSM) Lille-Métropole, Lille, France; 6 Service de psychiatrie, Centre hospitalier de Cayenne, Cayenne, French Guiana; PLOS: Public Library of Science, UNITED KINGDOM OF GREAT BRITAIN AND NORTHERN IRELAND

## Abstract

French Guiana is a culturally diverse and socioeconomically heterogeneous territory. We aimed to describe social representations of madness, mental illness, and depression in the general population of the Cayenne area and to explore similarities and differences across sociodemographic subgroups. Cross-sectional data from the 2021 Mental Health in General Population survey were analyzed (N = 881 adults). Open-ended responses to definitions of “mad person,” “mentally ill person,” and “depressed person” were thematically coded. Perceptions of behaviors, causes, recognition, responsibility, suffering, treatability, and care pathways were assessed. Multivariate generalized estimating equations examined associations with sex, age, education, income, social situation, and country of birth. Representations distinguished depression (linked to sadness, life events, suffering) from madness and mental illness (overlapping, associated with loss of control, danger, brain dysfunction). Violence and delusions were attributed to madness/mental illness; emotional withdrawal to depression. Life events were the leading perceived cause across conditions, with biomedical views for mental illness. Professional help-seeking was widely endorsed, though hospitalization for madness notably. Stigma followed a hierarchy: highest for madness, lowest for depression. Thematic representations were homogeneous across subgroups. Subgroup differences emerged mainly in perceived dangerousness, abnormality, care preferences, and child inclusion, driven by socioeconomic factors (lower education/income associated with higher perceived danger and less formal care endorsement) rather than country of birth. Contrarily to our initial hypothesis that the diverse origins of the population would translate in heterogeneous representations in our sample, shared representations predominated, with socioeconomic inequalities shaping variations in stigma and attitudes more than cultural origins.

## Introduction

Although mental health is more openly addressed than before, stigma towards psychiatric diseases remains widespread. Studies show that cultural and sociodemographic factors shape mental health representations, with implications for stigma [1–3], help-seeking [1,4], and treatment efficacy. Cultural frameworks (collectivism vs. individualism, religious beliefs) influence how mental illness is perceived and expressed, while sociodemographic factors (education, income, ethnicity) drive disparities in access and attitudes [1,4–6]. It is however often impossible to disentangle true associations from the multiple potential confounders [7].

The Mental Health in the General Population (MHGP) surveys were conducted in mainland France and all overseas territories, except Saint-Pierre-and-Miquelon and Wallis-and-Futuna. The surveys, designed and coordinated by the WHO collaborating center in Lille, aimed to provide quantitative estimates of the prevalence of different mental health issues and their risk factors [8,9], but it was also preoccupied by the promotion of mental health and reduction of stigma [10,11]. The population’s knowledge of mental health is linked with attitudes towards those thought to have a mental disorder. Although knowledge in the population is often good overall, there is much heterogeneity of lay knowledge, discourses, behaviors, and attitudes concerning mental health. Hence, negative stereotypes shaped by local cultures remain strongly attached to the mentally ill.

The MHGP surveys allowed to screen the images of “depression”, “mental diseases”, and “madness” in the general populations of culturally varied French territories. Somewhat counterintuitively, the social representations of the “madman”, the “mentally ill”, and the “depressive” in the Overseas Territories and in Mainland France generally showed no fundamental differences, consistent with results found in other national MHGP survey sites [12–14]. At first glance, they appeared constant and homogeneous. The main initial explanatory hypotheses were that social representations differ by category (madness, mental disorders, versus depression), that some are culturally invariant, archaic, whereas others are culturally variable and evolving. MHGP studies hence inform anti stigma campaigns through the study social representations and the analysis of exclusionary attitudes. The mad person representation was relatively stable and universal across cultures, whereas representations of the depressed person vary according to specific cultural and social contexts. The study also hypothesized that the social representation of a disorder determined the level of exclusion or identification. The more a condition was linked to insanity, the higher the fear, dehumanization, stigma and social exclusion; the more it was linked to familiar life problems –i.e., depression— the easier it is for individuals to seek help. The category mentally ill represented a medicalized transition between insanity and depression but remained associated with exclusion and danger. Such representations influence exclusion, fear, and help-seeking attitudes.

Across all sites, the insane person was invariably consistently viewed as dehumanized, defined by a deficiency (lack of reason/control) and perceived as dangerous and unpredictable. By contrast, there was more variability regarding the view of the depressed person. In France, depression was associated with fatigue, sadness, and external emotional shocks whereas in New Caledonia’s Kanak Community, the representation of depression was less individualized and often overlapped with the image of the insane person, with an explosive and violent image. In Polynesia, representations of depression and mental illness appeared more deeply intertwined with collective, spiritual, and relational dimensions [14]. However, despite the overall apparent similarity of representations in the complete samples of each territory there may have been significant differences between subgroups within territories.

French Guiana is a French overseas territory lying between Brazil and Suriname in South America. Despite equality of rights, the social structure and the view of institutions remains influenced by its colonial history. Indigenous Amerindian peoples, Afro-descendant communities and more recent migrant populations are often more affected by structural inequalities. Half of its adult population consists of immigrants often with little education and low health literacy, over half of the population lives below the poverty level of mainland France, and 29% live in great poverty. The cost of life is substantially greater than in mainland France (34% for food, 15% for manufactured products, 20–35% for housing), which further burdens the large proportion of the population living with low incomes.

Given these characteristics, we hypothesized that the images of madness, mental diseases, and depression may differ between population groups. Our objectives were thus to describe these perceptions in the general population in the Cayenne area, and to compare them between different subpopulations. Such information was assumed to help optimize communication against, and prevention of, stigmatizing attitudes towards mental health problems.

## Methods

### Ethics statement

The study complies with the Declaration of Helsinki. It was approved by the Comité Consultatif sur le Traitement de l’Information en matière de Recherche dans le domaine de la Santé (CCTIRS) (No. 98.126). All participants provided written informed consent.

### The Survey: “Mental Health in the General Population” (MHGP)

This program was initiated in 1999 by the WHO Collaborating Centre (WHOCC). The survey was deployed in mainland France and its overseas territories, including French Guiana. The methodology is detailed in reference [11].

We analyzed data from the cross-sectional survey conducted in the coastal municipalities of French Guiana. The inclusion criteria were: sufficient command of French, Creole, English or Portuguese, informed consent, age > 18 years, and not residing in an institution or experiencing homelessness.

Quota sampling was used to obtain a representative population by age, sex, education level, and occupation, based on 1999 INSEE census data [15].

### Study sample

The survey was conducted between 01/03/2021 and 31/08/2021 in six municipalities (Cayenne, Macouria, Matoury, Roura, Montsinéry-Tonnégrande, Rémire-Montjoly). These municipalities do not include Western French Guiana, Eastern French Guiana, or the inland municipalities, which are culturally different from the central coast. Furthermore, the languages used did not include the Maroni River languages or Amerindian languages. Nine hundred individuals over the age of 17 were interviewed face-to-face by trained interviewers in French, Guianese Creole, Portuguese, or English. Ultimately, 881 complete questionnaires were analyzed.

### Tools used

#### Representations of “madness”, “mental illness” and “depression”.

The interviewers (nurses and psychologists) were trained by WHOCC experts. The survey began with four open-ended questions: 1) In your opinion, what is mental health? 2) In your opinion, what is a mad person? 3) In your opinion, what is a mentally ill person? And 4) In your opinion, what is a depressed person? The last three questions were also asked again at the end of the questionnaire on representations of mental health. Textual analysis was conducted on responses translated into French. These short responses were thematically coded (S1 Table).

The sociodemographic variables analyzed included: age, sex, education level, marital status, professional activity, and monthly household income categories.

**Statistical analysis.** After simple descriptive statistics of the sample characteristics, the responses showing the representations of different mental health terms were described. Response distributions were summarized as percentages and represented as synthetic bar charts. Broad themes were coded in open-ended definitions (theme mentioned yes/no) using the codebook in S1 Table. A multiple correspondence analysis was performed using the themes extracted (1 = present and 0 = absent). The Kaiser rule (retaining dimensions with eigenvalues >1) was used to retain the main components.

Our aim was to evaluate the general impact of covariates on the overall survey behavior. Thus, to assess whether attitudes and beliefs varied across sociodemographic subgroups, we fitted pooled logistic regression models using generalized estimating equations with the assumption of an exchangeable correlation structure. Within each domain, multiple binary questionnaire items were analyzed together using population-averaged logistic generalized estimating equations (GEE) models. Data were reshaped into a long format so that each observation corresponded to a single item response, and item fixed effects were included to control for differences between questions. Clustering at the respondent level accounted for correlations among responses from the same individual. The resulting estimates therefore reflect average associations across items within a domain providing a synthetic measure of overall response patterns taking into account within-respondent correlation [16]. Models adjusted for sex, age group, income, education level, social situation, and country of birth. The reference groups for the Adjusted Odds Ratios (AOR) were respectively: female, youngest age group, lowest income category, lowest education level, stable/working social situation, and born in French Guiana. Because a large number of adjusted associations were evaluated across outcomes and sociodemographic categories, we additionally controlled for multiple comparisons using the Benjamini–Hochberg false discovery rate (FDR) procedure applied to all p-values from the adjusted GEE models. Forest plots were computed. Data analysis was performed with STATA 19 and python 3.10.

## Results

Sociodemographic characteristics of the study population (Table 1).

**Table 1:** Sociodemographic characteristics of the study population (French Guiana). N = 881.

Characteristic	Category	%
Gender	Female	53.0
	Male	47.0
Age (years)	18–29	25.4
	30–39	21.5
	40–49	20.5
	50–59	16.2
	≥ 60	16.3
Marital status	Single	47.8
	Married/ cohabiting/ civil partnership	42.7
	Separated/ divorced	4.7
	Widowed	4.8
Educational level	No schooling	9.3
	Incomplete primary education	16.2
	Completed primary education	17.7
	Incomplete secondary education	17.3
	Completed secondary education	17.8
	University level or equivalent	21.8
Employment status	Employed	59.2
	Not employed	40.8
Monthly household income (€)	< 840	24.3
	840–1,300	13.9
	1,300–2,520	32.4
	> 2,520	29.4
Country of Birth		
	French Guiana	43.4
	France	22.8
	French Antilles	7.1
	Haiti	19.7
	Brazil	5.7
	Other	1.3

In the surveyed population, more than half of respondents were women (53%). The mean age of respondents was 42.3 years (SD = 15.8). Single individuals represented nearly half of the sample. Over 43% of the sample had at most primary education. Individuals reporting a monthly household income below €1,300 represented 38.2% of respondents (Table 1).

The mean number of persons per household was 3.6 (SD = 3.2). The population was predominantly religious (77.6%), although only 51.9% reported practicing their religion. The dominant religion was Catholicism (44.5%). More than four out of ten respondents were first-generation migrants (41.5%).

### Representations: Descriptive analysis

**Fig 1:**
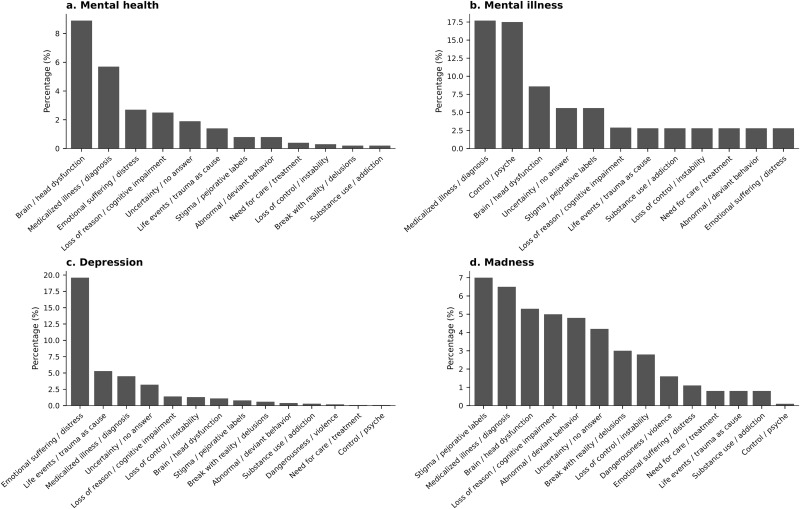
Thematic analysis of the representations of the concepts of mental health, mental illness, depression, and madness in the general population of the Cayenne area, French Guiana.

*Thematic analysis*. The ranking of the thematic codes extracted from the thematic analysis shows the difference between the 4 vignettes mental health, mental illness, depression, and madness with depression standing apart from the other vignettes with a greater emphasis on suffering and life events than the other vignettes where medical, cerebral, or genetic issues are put forward by the respondents, with stigma as a major theme for madness (Fig 1a, 1b, 1c, 1d).

The words most strongly associated with “mental health” were: health, well-being, physical, balance, state, psychic, and psychological. For “depressed,” the most representative words were: cry, sad, depression, tired, negative, and emotion. The definitions of “mad” and “mentally ill” shared a high degree of similarity. These analyses thus indicate that respondents clearly distinguished depression, mental health, and mental illness. However, representations of mental illness remain very close to those of madness.

### Multiple correspondence analysis

As commonly observed in MCA of social representations, inertia showed a gradual decay distributed across several dimensions, reflecting a high degree of shared representations in the population. Based on Kaiser’s rule, the three retained dimensions captured approximately 30% of the total variance in the thematic representations of mental health concepts. They structure the respondents’ discourse as dimension 1 along a medical—social moral axis (12% of variance): High loadings refer to medicalized illness, brain/ head dysfunction, and diagnosis/ treatment which are opposed to stigma/pejorative labels, and abnormal or deviant behavior. Dimension 2 (10% of variance) was spread along a fear—empathy axis, captured dangerousness/ violence, loss of control, and break with reality opposed to emotional suffering and life events/ trauma. Finally, dimension 3 (8% of variance) spread along a cognitive rupture—psychosocial vulnerability axis captured loss of reason/ cognition, delusions, incoherence contrasted with trauma, stress, social adversity.

### Dangerous behaviors attributed to madness and mental illness

**Fig 2:**
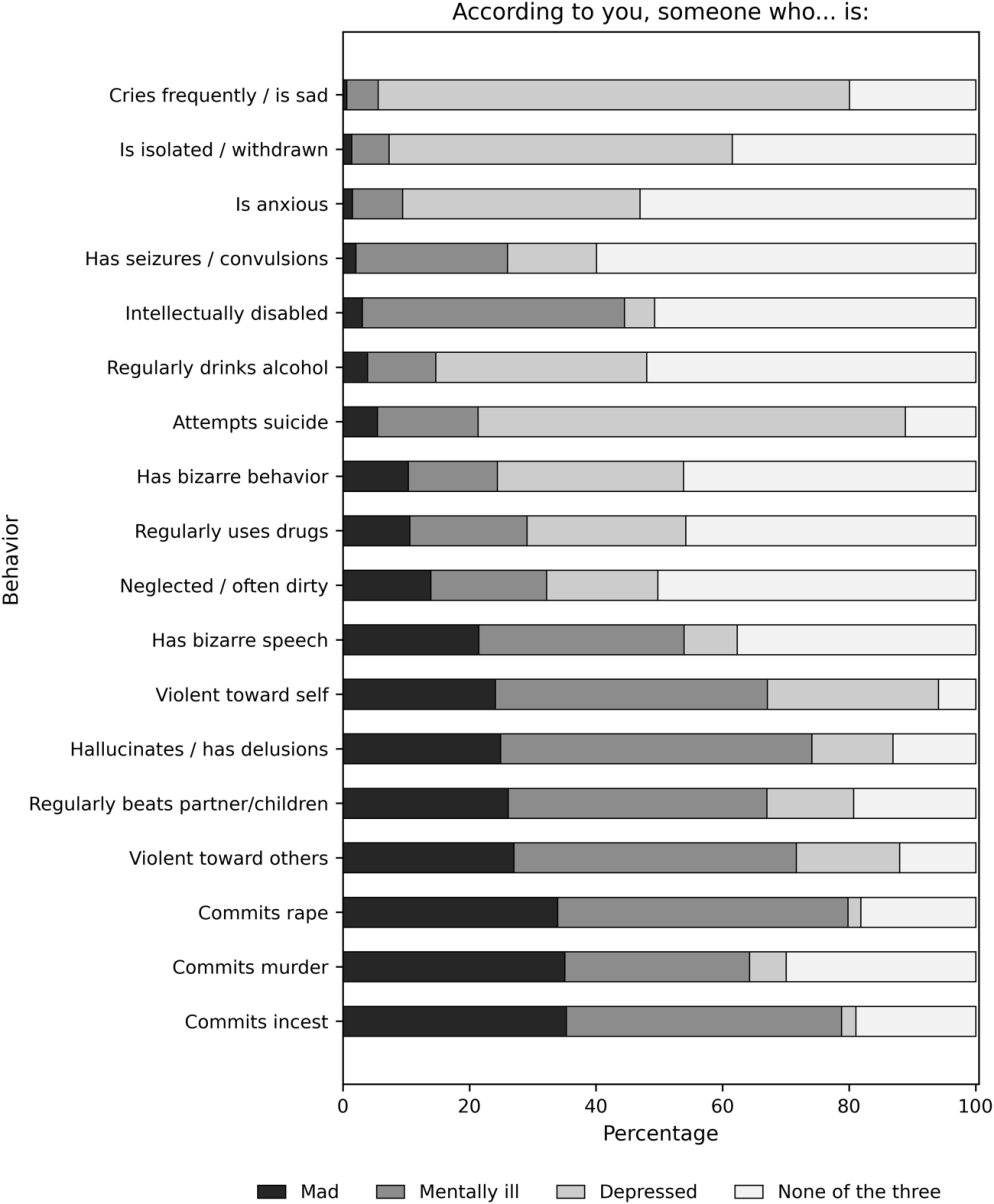
Frequency of behaviors attributed to someone who is “mad”, “mentally ill,” or “depressed”.

**Fig 3:**
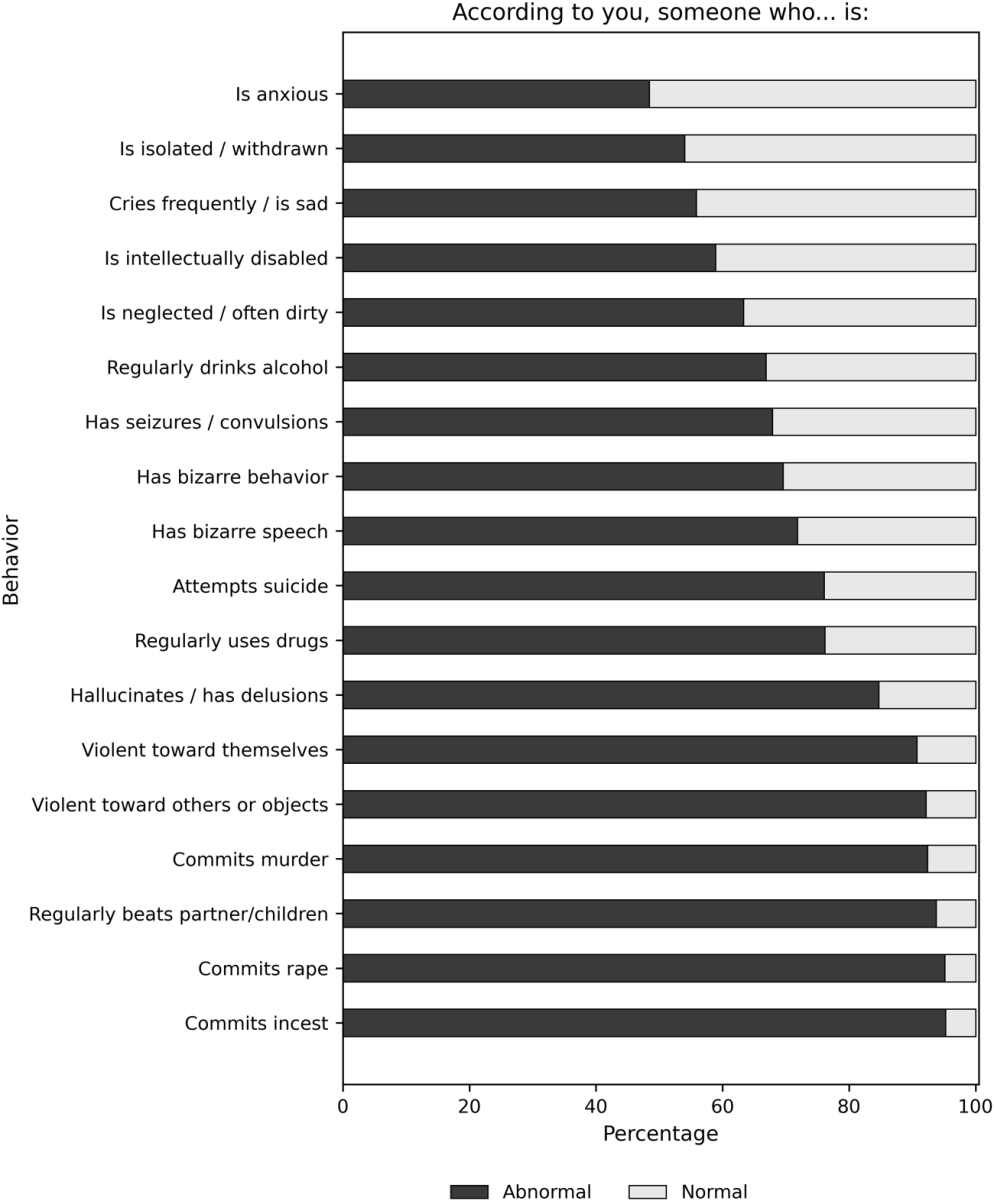
Judgment on the normality of behaviors of someone who is “mad”, “mentally ill”, or “depressed”.

**Fig 4:**
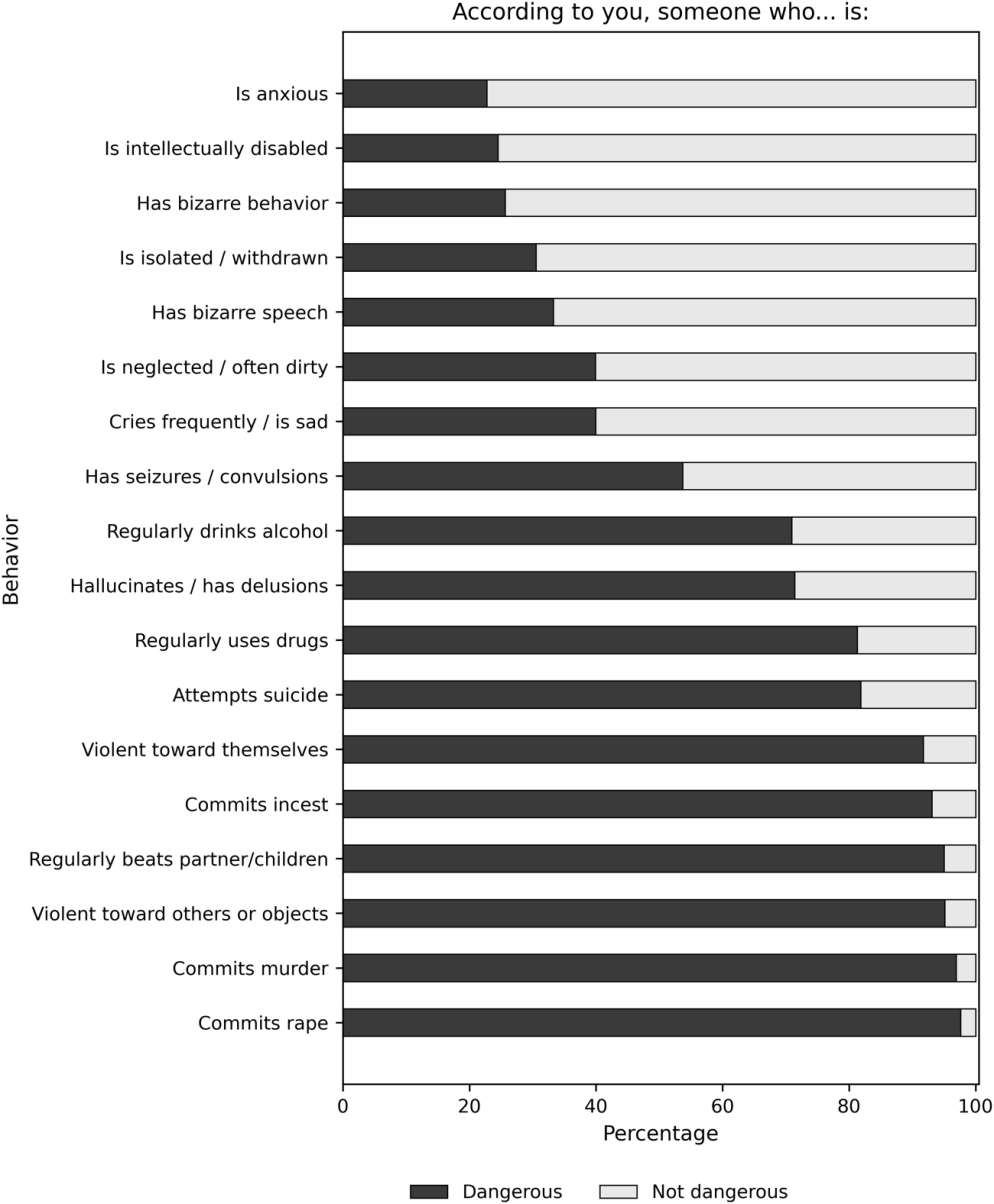
Judgment on the dangerousness of behaviors of someone who is “mad”, “mentally ill”, or “depressed”.

Fig 2, Fig 3 and Fig 4 show the representations about the behavior, perceived normality and dangerousness of mental health issues. Violent behaviors, such as committing incest, rape, or murder, were associated with madness by four out of ten respondents. For more than 40% of respondents, individuals are considered mentally ill when they are violent toward others or objects, hallucinate or commit rape, or regularly beat their spouse or children. More than 90% judged these behaviors to be abnormal and dangerous.

By contrast, behaviors such as frequent crying, sadness, suicide attempts, isolation, and withdrawal were mainly associated with depression. These behaviors were generally considered abnormal but less dangerous.

Behaviors such as intellectual disability, anxiety, neglect, poor hygiene, isolation, bizarre speech, and unusual behavior were infrequently attributed to madness or mental illness and were not perceived as dangerous.

### Recognition of “madness”, “mental illness”, and “depression”

For the population of the six municipalities of the CACL, madness, mental illness, and depression were primarily recognized through behavior (S1 Fig) Madness and mental illness were then identified by speech and appearance, whereas for depression the inverse pattern was observed.

### Perceived origins of mental illness, depression, and madness

Conditions labeled as madness were more frequently attributed to intrinsic or irreversible causes, whereas depression was more often associated with psychosocial or situational factors S2a–S2c Fig shows the perceived causes for the 3 vignettes. Across all three vignettes, life events were the most frequently cited cause, particularly for depression and madness. Physical causes were most frequently cited for mental illness, exceeding all other categories. Addictive origins were commonly mentioned across the three conditions, while magico-religious explanations and divine causation were rarely reported. Social and socio-economic factors were less frequently cited than individual-level explanations, regardless of vignette. Very few respondents mentioned the COVID-19 pandemic as a cause of depression, madness, or mental illness.

### Perceptions of responsibility, suffering, and exclusion

**Fig 5:**
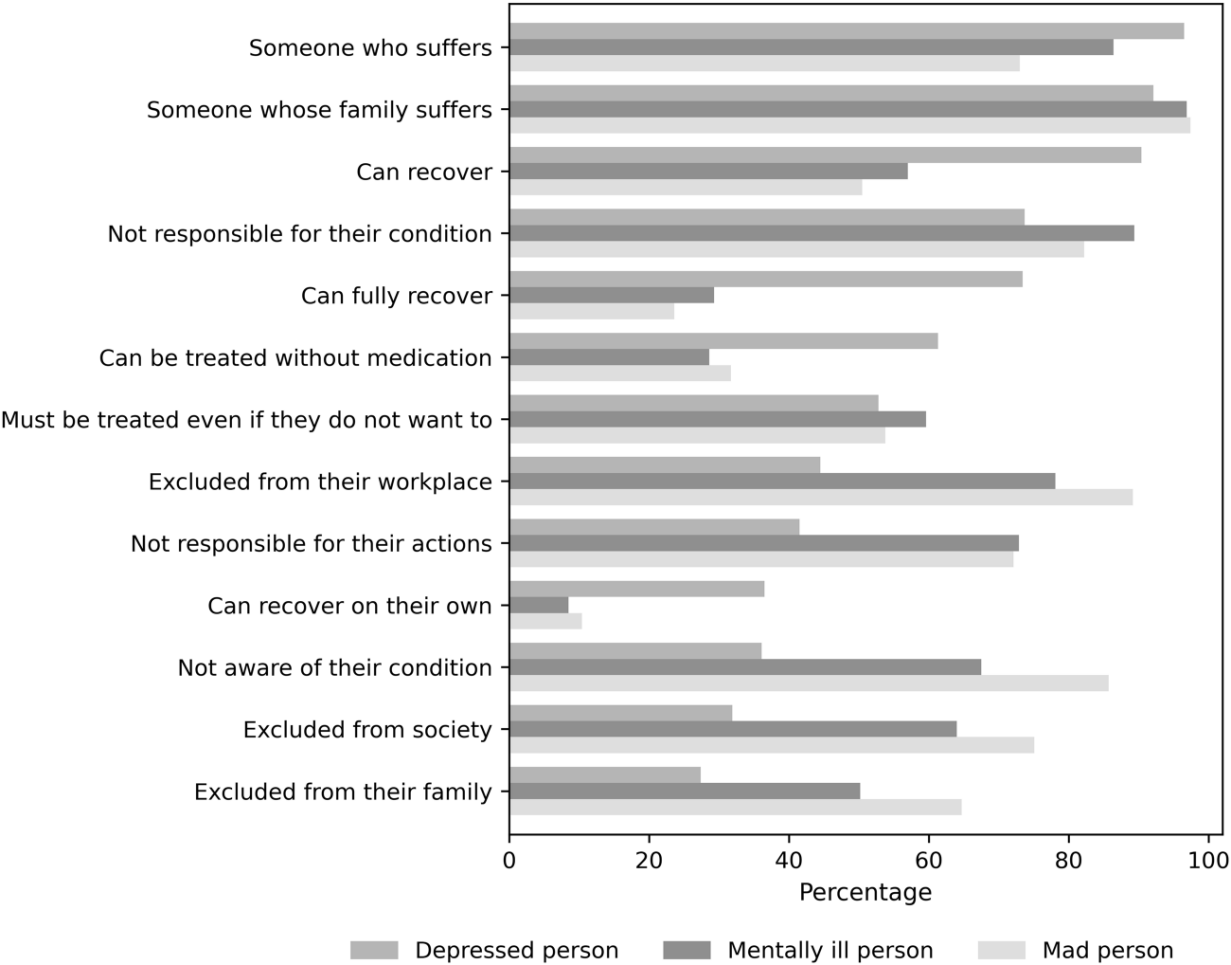
In your opinion, a “mad person”, a “mentally ill person”, and a “depressed person” is….

Fig 5 shows that the majority of respondents considered that a mad person or a mentally ill person is neither responsible for their actions nor for their condition, and that the person is not aware of it. A mad person was perceived as suffering less than a mentally ill person, who was perceived as suffering less than a depressed person. A similar gradient was observed for family suffering and social exclusion. More than half of respondents considered recovery possible for madness and mental illness, but fewer believed in complete recovery. In contrast, nearly nine out of ten believed that a depressed person could recover, and more than seven out of ten believed in complete recovery. Compulsory treatment was considered justified by more than half of respondents for all three conditions.

### Treatability of mental health issues

**Fig 6:**
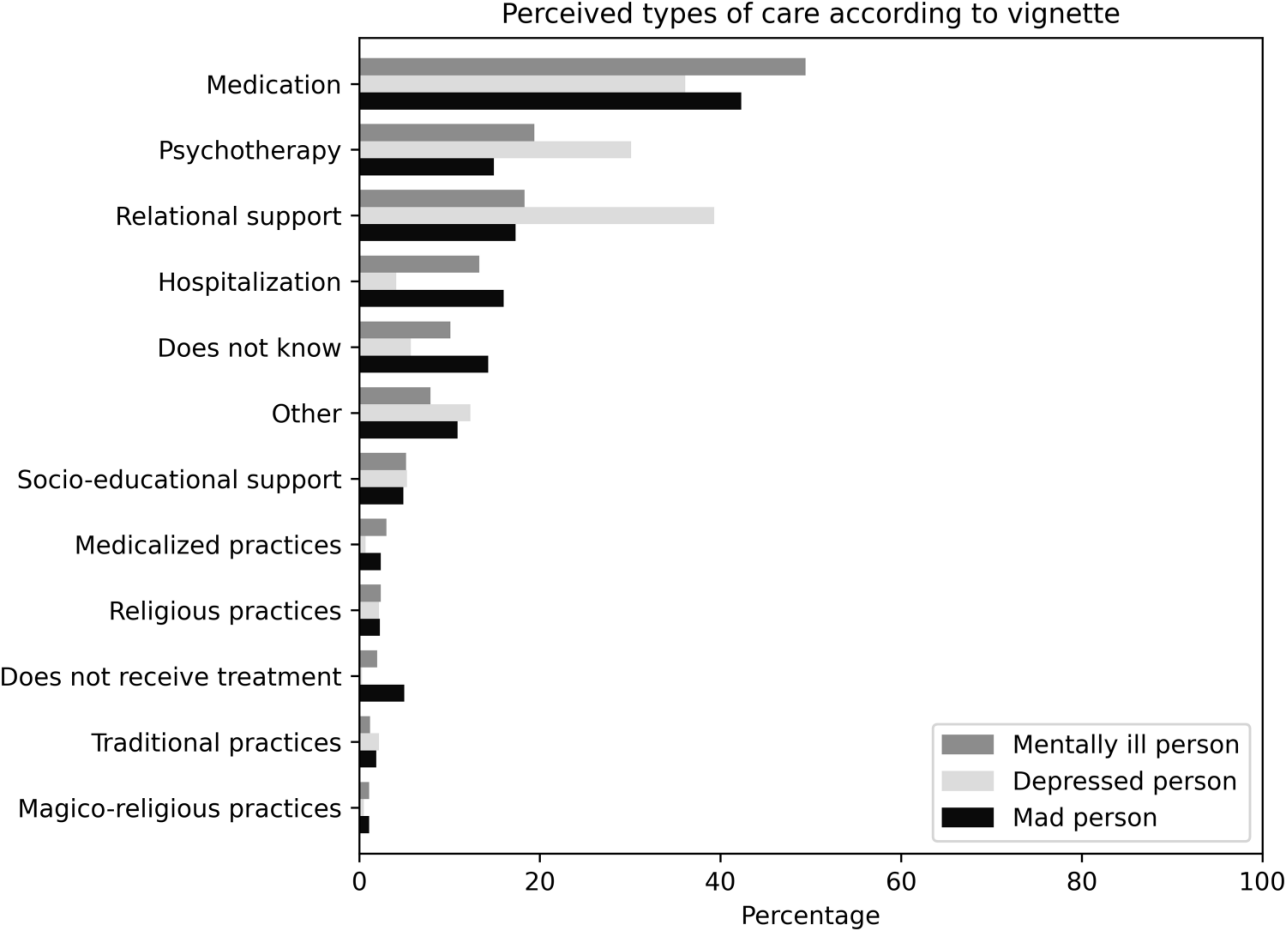
In your opinion, how can a “mad person”, a “mentally ill person”, or a “depressed person” be treated?.

Fig 6 shows the responses regarding the treatability of mental health issues.

### Care and help-seeking pathways

More than 85% of respondents would advise a close relative—whether mad, mentally ill, or depressed—to consult a mental health or general health professional (S3 Fig). The social network was the most frequently cited first source of help for oneself. Medication was the most frequently cited treatment for madness and mental illness, whereas relational support was most often cited for depression (S4 Fig). Hospitalization was more strongly associated with madness. More than 12% of respondents believed that madness could not be treated.

Respondents expressed differentiated representations of madness, mental illness, and depression, with consistent gradients in perceived causes, treatability, recovery, and social proximity. (S5 Fig). Correspondingly, respondents were less likely to consider that a “mad person” could be treated without medication, recover fully, or recover spontaneously, while these possibilities were endorsed substantially more often for depression and, to a lesser extent, for mental illness.

Beliefs about suffering followed a similar pattern. While the vast majority of respondents considered that individuals in all three categories suffer, perceived suffering of family members and expectations of social exclusion were markedly higher for madness than for depression. Support for compulsory treatment in the absence of consent was high–over half were favorable to it— in all three vignettes.

Finally, self-reported social proximity differed sharply across categories. Respondents were substantially more likely to report knowing someone who had experienced depression than someone described as mad or mentally ill. Overall, these results indicate that stigma-related beliefs are not uniform across mental health conditions but instead follow a structured hierarchy, with madness consistently perceived as less treatable, less recoverable, more socially distant, and more legitimate of coercive care.

### Perception of responsibility, awareness, suffering and exclusion

Overall, families were thought to suffer for all three representations, whereas for patients depression was most associated with awareness of their condition and suffering (S6 Fig). Depression was associated with the greatest responsibility for their actions, and their condition. Madness was most frequently associated with exclusion from family and society and were thought to suffer less and be least aware of their condition.

Attitudes toward care pathways, family involvement, and reintegration (S7 Fig and S8 Fig).

Across questions respondents expressed a predominantly family- and care-oriented approach to mental illness, with some differences according to the type of condition. Knowledge of alternatives to psychiatric hospitalization was more frequently reported for depression and mental illness than for madness, while psychiatric hospitalization was most often recommended for individuals described as mad and least often for those described as depressed. Acceptance of home care followed the same gradient, increasing from madness to depression.

Decision-making was largely collective: a majority of respondents indicated that they would organize a family meeting before making decisions for an affected relative, regardless of the condition. Most respondents believed that families could reintegrate relatives or children who had been treated for mental illness or depression, whereas acceptance dropped markedly for stigmatized conditions such as addiction and especially sexual violence.

Perceived burden at home was highest for relatives described as mad, intermediate for mentally ill, and lowest for depressed. Situations prompting recourse to external help were commonly linked to aggressive behavior, loss of control, or safety concerns, particularly for madness and severe mental illness. Finally, when advising help-seeking, respondents most often recommended healthcare professionals, although non-medical and informal resources remained frequently cited, especially for depression.

### Child-related attitudes

**Fig 7:**
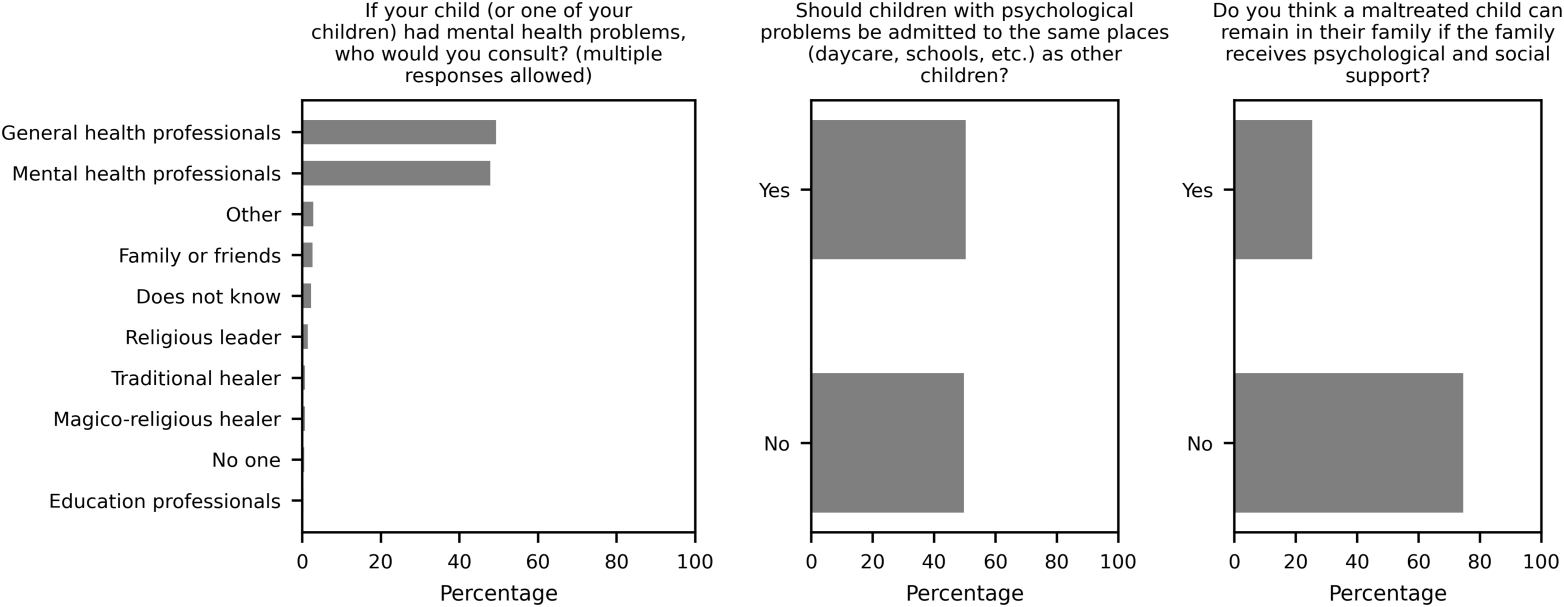
Child-related attitudes.

When asked whom they would consult if their child had mental health problems, respondents most frequently cited health professionals, particularly mental health professionals, followed by general health professionals and family or friends (Fig 7). Recourse to religious, traditional, or magico-religious figures was reported by a smaller proportion, while a minority indicated that they would consult no one or other sources. Regarding social inclusion, opinions were divided. Approximately half of respondents did not agree that children with psychological problems should be admitted to the same institutions (daycare centers, schools) as other “normal” children, indicating inclusion reticence.

Finally, a majority of respondents did not agree that a maltreated child should remain within their family, even if that family were to receive psychological and social support. This suggests a prevailing preference for protective separation rather than family preservation in situations of child maltreatment.

### Multivariate analyses looking at the impact of covariates

#### Thematic analysis.

In multivariable population-averaged models adjusting for sex, age group, country of birth, income, social situation, and education level, no sociodemographic characteristic was significantly associated with the likelihood of expressing any thematic code for “madness”, “mental illness”, or “depression”. This absence of association was consistent across all vignettes and thematic domains. The semantic structure of spontaneous representations was stable across sociodemographic groups, suggesting shared narratives about mental illness when respondents are prompted to explicitly describe their perception.

To avoid over-interpretation of isolated findings, we present a comprehensive summary table (S2 Table) including all adjusted estimates—both significant and non-significant—allowing readers to assess the overall pattern of results. Across the full set of GEE analyses we examined 365 adjusted associations covering multiple domains (representations of mental illness, stigma and dangerousness, responsibility and awareness, care, treatment, and reintegration, child-related attitudes). The dominant pattern showed that most odds ratios were close to 1, most confidence intervals included 1, and most associations were not statistically significant. (see S2 Table). Despite the identification of some statistically significant associations (26 of 365 adjusted comparisons across multiple pooled GEE models, outcomes, and sociodemographic categories) of moderate effect size, particularly related to income and education, the overall influence of sociodemographic variables was limited. Most adjusted associations were close to the null, suggesting that representations and attitudes toward mental illness were largely shared across the study population. Furthermore, after Benjamini–Hochberg FDR correction, only 4 associations remained significant at q < 0.05 (7 at q < 0.10). These associations were of moderate magnitude and were primarily related to socioeconomic characteristics, reinforcing the conclusion that sociodemographic differences were limited overall and that most perceptions and attitudes were broadly similar across sociodemographic groups.

### Synthetic overview

S9 Fig shows adjusted population-averaged odds ratios from generalized estimating equation models pooling all questionnaire outcomes. Displayed with 95% confidence intervals are all associations that remained statistically significant after false discovery rate correction, together with the largest observed contrast per sociodemographic predictor. Overall, few subgroup differences were observed, and effect sizes were modest, with socioeconomic indicators showing stronger associations than country of birth. Because a large number of associations were tested, we present two complementary summary figures. The combined forest plot (S9 Fig) shows both false-discovery–rate–significant associations and the largest observed contrasts per sociodemographic predictor, providing an overview of effect sizes. A second, more conservative forest plot (S10 Fig) displays only the strongest remaining associations after false discovery rate correction. The contrast between these figures illustrates that most subgroup differences are modest and do not remain statistically robust after correction for multiple testing.

## Discussion

The MHGP study is the first study to provide data on the representations of mental health in French Guiana. Our initial hypothesis was that in our culturally diverse territory there may be differences of representations of “madness”, “depression”, and “mental illness” between population subgroups within the general population of French Guiana. The sample was balanced with a third of persons being born in another country, with over 42% having at most primary education, with a range of incomes. Regarding religion, our sample (77.6% affiliated, 51.9% practicing) was broadly consistent with population-level estimates [17]. Subgroup analyses revealed that education, income, and social position did shape how individuals interpreted responsibility, danger, care, and inclusion, but the effect size was usually modest. In multicultural French Guiana, it is frequent to culturalize poverty and misattribute socioeconomically determined differences to culture. The present results, adjusting for such potential confounding, hence tend to downplay the differences in representations between country of birth, which is new and somewhat counter intuitive [1,3–5,7,18]. What was perhaps even more striking was that, overall, there were great similarities between groups, a finding that echoes the homogeneity of representations between overseas territories [13]. Mental illness was hence understood in broadly similar ways across social groups, suggesting shared representations at the population level. Socioeconomic factors—more than cultural origin—were associated with differences in stigma, responsibility, and attitudes toward care and inclusion. These findings imply that stigma-reduction strategies should prioritize social inequalities and structural determinants rather than relying on culturally segmented interventions.

The terms used in the survey of representations voluntarily explored lay terms (madness) which is a crude phenotypic description, a popular interpretation of delusions and medical terms (depression and mental illness) which elicit different responses. The analysis of open-ended definitions shows that respondents clearly distinguished depression, mental health, and mental illness, while representations of madness and mental illness largely overlapped. Depression was primarily associated with sadness, crying, emotional distress, and difficulties related to life events, whereas mental health was mainly defined in terms of balance, well-being, and psychological or physical equilibrium. In contrast, madness and mental illness were frequently described using similar lexicons referring to pathology, brain dysfunction, loss of control, abnormal behavior, and detachment from reality. It is possible that the terms chosen mobilized ideas and representations circulating widely within French territories, French cultural representations about important phenomenon percolating through population subgroups and consolidating into social norms that are relatively compatible across the population. By contrast, the overall homogeneity between territories and population subgroups could imply more archaic human universals (normal vs. abnormal states, actions under self-control or not in control…) [19]. Our results suggest that both perspectives may be pertinent when surveying populations about mental illnesses.

Behaviors involving violence, criminal acts, and loss of contact with reality were predominantly attributed to madness and mental illness, while they were rarely associated with depression. Respondents reported that behavior was, by far, the primary feature used to recognize a person described as mad, mentally ill, or depressed, followed by speech and, to a lesser extent, appearance. Most respondents considered individuals described as mad to be less responsible for their actions, less aware of their condition, and more likely to be excluded from family, work, and society. Individuals described as depressed were substantially more often viewed as responsible for their actions and aware of their condition, and far less frequently perceived as socially dangerous.

The perceived origins differed across the three conditions: 1/Depression was mainly attributed to life events and addictive behaviors, followed by mental health–related causes and relational difficulties. 2/ Mental illness was predominantly associated with physical origins, life events, and addictions, suggesting a more biomedical representation. 3/ Madness was also frequently attributed to life events and addictions, but with a broader dispersion of perceived causes, including mental health and physical origins. Across all three, supernatural or religious explanations were rarely cited, which counters the frequent circulation of anecdotal reports accentuating salient cultural differences.

When asked whom they would advise a relative to consult, respondents overwhelmingly recommend health professionals, both general health professionals and mental health specialists, regardless of whether the relative is described as “mad”, “mentally ill”, or “depressed”. Social relations are cited as a secondary resource, particularly for depression. Religious, magico-religious, or traditional healers are mentioned far less frequently, and very few respondents indicate that no help should be sought. There again, the relatively rare frequency of advising traditional or magico-religious healer to one’s relative countered our expectations as anecdotes on such practices circulate widely among health professionals and non-government associations. Ranking care options by overall frequency hence seemed to confirm a preference for formal medical and psychiatric care, indicating broad social acceptance of biomedical approaches to mental health problems, despite the persistence of stigmatizing representations of “madness” and “mental illness”. However, despite this apparent preference, a recent analysis of the same sample showed that, in practice, persons with depression and post-traumatic stress disorder rarely consulted and often resorted to traditional healers [8,20]. But in fact those who sought medical car were also more likely to seek traditional care. This discrepancy between declared biomedical norms and actual therapeutic itineraries reflects therapeutic pluralism: the coexistence of biomedical and traditional care systems within individual health-seeking trajectories, rather than a simple opposition between them, as described in French Polynesia [14,21]. Social desirability may push respondents to align declared preferences with biomedical norms. The gap between declared and practiced care reflects a tension between public discourse and private therapeutic behavior.

Psychiatric hospitalization was frequently endorsed for the “mad” and “mentally ill” vignettes, while support for hospitalization was more ambivalent for “depression”. Home-based care was more acceptable for “depression” than for the other conditions. Knowledge of alternatives to psychiatric hospitals was limited overall, especially for the “mad” vignette, suggesting a narrow conception of available care options. Family deliberation before decision-making was commonly endorsed across conditions, but acceptance of reintegration into the family varied substantially depending on the condition and its perceived dangerousness. The construction of lay representations of “madness” is inseparable from the institutional apparatus of psychiatry and its aura within the community where the survey took place. During the 20^th^ century there were no psychiatrists in French Guiana until 1970 and patients, when they were not imprisoned, were either managed by non-psychiatrists, or evacuated towards specialized facilities in Guadeloupe or Martinique. Since the 1980s, In Cayenne, psychiatric inpatient care is concentrated in a single unit at the Centre Hospitalier de Cayenne, which has housed a dedicated psychiatric ward. The number of psychiatric beds per capita remains significantly lower than in mainland France.

Regarding “mental illness” in children, it was striking that nearly half of the surveyed population did not agree that children with psychological problems should attend the same institutions as other children. Inclusivity of institutions towards children with psychological difficulties is a pillar of care [22]. It is not clear if this split finding reveals widespread stigmatizing attitudes or if it reflects an implicit belief that such children should benefit from more appropriate institutions for their needs.

The present study has a number of limitations. First, the sample concerned Creole, English, Portuguese, or French-speaking persons living in the Cayenne area, which is a restricted part of the complex patchwork of languages and cultures in French Guiana. Hence, for example, Amerindians, Maroons, living in the interior were hardly sampled. Although a third of the sample was born in a foreign country, it may not have been sufficient to identify cultural differences between countries of birth. Importantly, the MHGP instrument did not include any variable capturing ethnoracial identity. The survey’s tools follow French regulation that require statistical color-blindness (because of the memory of Nazism and ethnic statistics) thus limiting the study’s capacity to precisely examine ethnic differences in mental health representations. The variable country of birth was only an imperfect and reductive proxy.

The cross-sectional declarative design does not allow to infer causality and may be affected by biases. The open responses remain short and do not allow in-depth analysis of beliefs and attitudes and should be ideally complemented by qualitative studies. Responses reflect social representations, not actual behaviors, and may be influenced by social desirability, for example by underreporting certain traditional beliefs and practices. In addition, the use of predefined vignettes necessarily simplifies complex clinical realities. However, this is the first study to actually provide data on this territory and it does so using the same design as other French territories and thus allowed interesting comparisons between a variety of cultures [5]. This study combines rich descriptive data with multivariable analyses accounting for repeated measures and vignette structure, allowing robust inference.

## Conclusion

We found that mental illness was understood in broadly similar ways across social groups, suggesting shared representations at the population level. However, some statistically significant differences of modest effect size were observed. These differences were more strongly associated with socioeconomic factors than with cultural origin, particularly with respect to stigma, responsibility, and attitudes toward care and inclusion.

## Supporting information

S1 Table Thematic coding of the responses to the vignettes. (DOCX)

S2 Table Comprehensive summary table including all adjusted estimates—both significant and non-significant. (XLSX)

S1 Fig In your opinion, how can we recognize a “mad person”/ “mentally ill person”/ “depressed person”?. (TIF)

S2 Fig a, b, c: Responses to the question “In your opinion, what can cause mental illness(a), depression(b), and madness(c)?”. (TIF)

S3 Fig If one of your relatives is “mad”, “mentally ill”, or “depressed”, who would you advise them to consult?. (TIF)

S4 Fig How can you treat them?. (TIF)

S5 Fig Representations regarding treatment and recovery of mental illness, depression, and madness. (TIF)

S6 Fig Perception of responsibility, awareness, suffering and exclusion. (TIF)

S7 Fig Attitudes before family reintegration. (TIF)

S8 Fig Attitudes about family reintegration, burden, and help-seeking. (TIF)

S9 Fig Trimmed synthesis of sociodemographic differences across pooled outcomes. This forest plot displays, for each sociodemographic variable, the single adjusted odds ratio furthest from the null value (OR = 1) observed across all modeled outcomes. Reference groups were age 18–29 years, primary education or less, male sex, and birth in France. Odds ratios were estimated using population-averaged logistic GEE models with fixed effects for items and adjustment for sex, age group, income, education level, social situation, and country of birth. Confidence intervals are shown on a logarithmic scale. This figure illustrates the maximum observed deviation from the null for each predictor and does not reflect the frequency or consistency of associations across outcomes. Each line represents an adjusted comparison between a sociodemographic subgroup and its reference category across pooled outcomes. Black symbols highlight associations that remain statistically significant after correction for multiple testing, while grey symbols show the largest observed contrasts even if not statistically significant after correction.
(TIF)

S10 Fig Strongest remaining associations after false discovery rate correction. After pooling outcomes, adjusting for all covariates, and correcting for multiple comparisons, only a very small number of sociodemographic differences remain, and these were modest in magnitude, and more often related to socioeconomic position than to cultural or national origin. This reinforces the central conclusion that shared representations dominate across groups, with limited, context-specific variation.
(TIF)

S1 Checklist Inclusivity in global research. (DOCX)
