# Supplementary material for: Representations of mental health, madness, mental illness and depression in the general population of French Guiana: Similarities and differences between sociodemographic subgroups
Source: PLOS Ment Health. 2026 Aug 13;3(8):e0000655. doi: 10.1371/journal.pmen.0000655 (PMC13472403; doi:10.1371/journal.pmen.0000655)
Supplement: Supplementary file 1 [file pmen.0000655.s001.docx]

**Table S1. Thematic coding of the responses to the vignettes**

| Code | Theme | Short Definition | Key Examples |
| --- | --- | --- | --- |
| 1 | Medicalized illness / diagnosis | Psychiatric framing as illness, pathology, or diagnosed disorder | “A person suffering from a mental illness”; “diagnosed… mental disorders”; “schizophrenia, bipolar disorder…” |
| 2 | Brain/head dysfunction | Problem located in the brain, neurons, or cerebral function | “Illness of the head”; “problem in the brain / neurons”; “cerebral / neuronal dysfunction” |
| 3 | Loss of reason / cognitive impairment | Incoherence, inability to reason or think clearly | “Inability to reason”; “doesn’t have all their mental faculties”; “irrational / incoherent” |
| 4 | Loss of control / instability | Failure to self-regulate emotions or actions | “Loses control… can’t manage”; “might snap”; “not master of their own mind” |
| 5 | Break with reality / delusions | Delusions, hallucinations, living detached from reality | “Loss of reality… delusional”; “lives in another world”; “sees things” |
| 6 | Abnormal / deviant behaviour | Violation of social norms, seen as bizarre or not normal | “Does the opposite of normal things”; “bizarre behaviour”; “doesn’t act according to society’s codes” |
| 7 | Dangerousness / violence / threat | Perceived risk of harm to self or others | “Someone dangerous”; “might kill… harm others”; “capable of… holding someone hostage” |
| 8 | Stigma / pejorative labels | Direct insulting or moral judgment via labels | “Mad”, “crazy”, “nutcase”; “He’s a psychopath”; “the ultimate madman” |
| 9 | Life events / trauma as cause | Explanation rooted in adversity, grief, or childhood experiences | “Painful events… emotional shock”; “childhood / family problems”; “bereavement, breakup…” |
| 10 | Emotional suffering / mal-être | Framing as sadness, deep unease, or depressive state | “Psychological suffering”; “feels unwell deep down”; “depressed… anxious” |
| 11 | Need for care / treatment | Emphasis on needing medical help, therapy, or institutionalization | “Needs treatment / therapy”; “should get treated / committed”; “followed by a psychiatrist” |
| 12 | Congenital/genetic | Seen as innate, hereditary, or “born with it” | “You’re born with it”; “genetic problem”; “born that way / hereditary” |
| 13 | Substance use / addiction as cause | Linked to alcohol, drugs, or addiction | “Smokes and drinks a lot of alcohol”; “drugs… crack”; “addiction” |
| 14 | Uncertainty / refusal | “Don’t know”, rejection of the concept, or difficulty explaining | “I don’t know”; “For me it doesn’t exist”; “Hard to explain” |
| 15 | Meta/critical reflection on the label | Comments on the term being outdated, pejorative, or socially constructed | “The term seems archaic”; “the word mad is pejorative”; “maybe we’re the mad ones” |
