# Supplementary material for: Representations of mental health, madness, mental illness and depression in the general population of French Guiana: Similarities and differences between sociodemographic subgroups
Source: PLOS Ment Health. 2026 Aug 13;3(8):e0000655. doi: 10.1371/journal.pmen.0000655 (PMC13472403; doi:10.1371/journal.pmen.0000655)
Supplement: Supplementary file 3 [file pmen.0000655.s015.docx]

**Manuscript Reference: PMEN-D-26-00039**

Journal: PLOS Mental Health

Date: June 2, 2026

**Title:** Representations of mental health, madness, mental illness and depression in the general population of French Guiana: similarities and differences between sociodemographic subgroups

# **Response to Reviewers**

Dear Editor and Reviewers,

We are grateful for the careful reading of our manuscript and the constructive comments provided by both reviewers. We have revised the manuscript accordingly and provide detailed point-by-point responses below. All changes are highlighted in the revised manuscript. We believe the revisions have substantially strengthened the work.

We begin with a general remark relevant to both reviewers' concerns: the MHGP survey instrument is a standardized tool used across all French territories, coordinated by the WHO Collaborating Centre in Lille. While we fully acknowledge its limitations — especially regarding the sampling frame, language coverage, and omission of certain community groups — the instrument was not designed by the authors of the present paper, and certain structural constraints (e.g., the absence of a social race variable, the exclusion of Maroon and Amerindian populations) are inherent to the study design. Our contribution lies in the analysis and contextualized interpretation of this dataset for French Guiana, for the first time. We have made this clearer in the revised manuscript.

**Response to Reviewer #1**

***Comment 1.1 — Social race, colonialism, and the positionality of the study***

*"The paper studies a French territory resulting from colonization. At no time is there any mention of questions of social race and colonization [...] In French Guiana, there are indigenous, black, white people... The interviewees have religious, spiritual, and spatially situated material considerations that are not only related to 'multiculturalism' but to relations of domination that should be questioned."*

We thank the reviewer for raising this important point. We fully agree that French Guiana is not simply a multicultural territory: it is a post-colonial space structured by historical relations of domination. Still today indigenous peoples, Afro-descendant communities (including Maroons and Creoles), and more recent migrant populations occupy very different positions in the social order, and these positions are shaped these historical dynamics.

However, we must be transparent about a key limitation of this dataset: the MHGP survey instrument does not include a variable capturing social race or ethnoracial identity. The closest available proxy is 'country of birth,' which is an imperfect and reductive measure of ethnoracial positioning. We have added an explicit discussion of this limitation in the revised manuscript. We note that this limitation is not unique to our study but reflects a broader blind spot in French public health research, where since the use of ethnicity by the Nazis, ethnicity is never an authorized variable, even if now it keeps ethnic inequalities invisible.

We have also added a contextual paragraph in the Introduction acknowledging the colonial history of French Guiana and its implications.

“Despite equality of rights, the social structure and the view of institutions remains influenced by its colonial history. Indigenous Amerindian peoples, Afro-descendant communities and more recent migrant populations are often more affected by structural inequalities. “

***Comment 1.2 — Contextualizing sample characteristics against the general population***

*"The average age of the participants, their salary, their relationship to religion, and gender representation are to be compared with the general population: what does 1300 euros per month represent in French Guiana? Is religion present in general or only in your sample?"*

This is an excellent point. We have added a new paragraph in the Methods sections contextualizing the key sociodemographic variables against available census and INSEE data for French Guiana.

Regarding income: the median monthly household income in French Guiana is substantially lower than in mainland France. Yet it is a French département and the cost of life is substantially greater than in mainland France (34% for food, 15% for manufactured products, 20 to 35% for housing) which makes it even more difficult for the poorest who have to pay more with meager incomes. We have specified this, thank you.

Regarding religion: the INSEE census for French Guiana indicates high rates of religious affiliation in overseas territories and in French Guiana. Our sample (77.6% affiliated, 51.9% practicing) is broadly consistent with population-level estimates, suggesting this is not a sample artifact. We have added this to the text.

***Comment 1.3 — Absence of the psychiatric institution and its social imaginary***

*"There is a lack of links with the psychiatric institution in French Guiana: what is the importance of psychiatric institutions in this territory? Since when have there been asylums and hospitals? Talking about madness and representations of madness without involving the psychiatric institution and its aura within the population is, in my opinion, damaging to the study."*

We agree that the relationship between lay representations of 'madness' and the psychiatric institution is important, and we thank the reviewer for pressing this point. The construction of the social imaginary of 'the mad person' is inseparable from the institutional apparatus of psychiatry — its history, its buildings, its potential coercive functions, and its aura in the community. Especially given the history of French Guiana.

We have added a new paragraph in the Discussion contextualizing the role of the psychiatric institution in French Guiana. *“The construction of lay representations of “madness” is inseparable from the institutional apparatus of psychiatry and its aura within the community where the survey took place. During the 20^th^ century there were no psychiatrists in French Guiana until 1970 and patients, when they were not imprisoned, were either managed by non-psychiatrists, or evacuated towards specialized facilities in Guadeloupe or Martinique. Since the 1980s, In Cayenne, psychiatric inpatient care is concentrated in a single unit at the Centre Hospitalier de Cayenne, which has housed a dedicated psychiatric ward. The number of psychiatric beds per capita remains significantly lower than in mainland France. “*

**Response to Reviewer #2**

We thank Reviewer #2 for their thorough reading of the manuscript and their detailed constructive suggestions. We have addressed each point in turn.

***Comment 2.1 — Overall assessment and MHPG methodology***

*"This article is clearly of scientific and public health interest and appears suitable for publication [...] One of its major strengths lies in challenging preconceived assumptions regarding cultural heterogeneity in French Guiana by demonstrating the predominance of shared representations across groups."*

=>thank you. We are grateful for this positive assessment. We agree with the reviewer's characterization of the study's main contribution and have sought to strengthen the framing of this core finding in the revised Introduction and Conclusion.

***Comment 2.2 — Traditional, cultural, and religious care: declared vs. actual practices***

*"The article therefore highlights an important distinction between declared public discourse and real-life therapeutic itineraries [...] social desirability bias may partly explain this result."*

We agree with the reviewer's interpretation. The discrepancy between declared preference for biomedical care and actual recourse to traditional healers (documented in a corollary study on the same sample) is a significant findings emerging from this dataset.

We have expanded the Discussion to make this tension more explicit, drawing on the concept of 'therapeutic pluralism' and the distinction between normative public discourse and private health-seeking behavior. We note that this gap likely reflects not merely social desirability bias, probable underreporting in survey contexts.

***Comment 2.3 — Comparison with MHPG surveys in French Polynesia and other overseas territories***

*"Given the limited number of studies on mental health in the general population in the French overseas territories, it appears important to cite and compare results not only with New Caledonia but also with the two articles concerning the French Polynesia MHPG survey."*

We thank the reviewer for this highly relevant suggestion and for providing the full references for the French Polynesia MHPG studies. What is described in Polynesia is indeed very relevant and similar to French Guiana.

We have added references to both Amadéo et al. (2022, L'Information Psychiatrique) and Amadéo et al. (2022, Journal of International Medical Research) in the revised manuscript.

***Comment 2.4 — Minor: CCTIRS approval number discrepancy***

*The two manuscript versions submitted contain slightly different CCTIRS approval numbers (No. 98128 vs No. 98.126).*

We apologize for this inconsistency, which arose during revision of the manuscript. The correct approval number is No. 98.126, as indicated in the supporting documentation (avis du CCTIR). We have standardized this across the manuscript.


We hope that these revisions adequately address the reviewers' concerns and that the manuscript is now suitable for publication. We remain available to provide any additional clarification or to make further changes at the Editor's request.

Sincerely,

The Authors
