# Supplementary material for: Representations of mental health, madness, mental illness and depression in the general population of French Guiana: Similarities and differences between sociodemographic subgroups
Source: PLOS Ment Health. 2026 Aug 13;3(8):e0000655. doi: 10.1371/journal.pmen.0000655 (PMC13472403; doi:10.1371/journal.pmen.0000655)
Supplement: Peer review history

# Peer review: Decision Letter 0

**DOI:** 10.1371/journal.pmen.0000655.r001

**Authors:** Karli Montague-Cardoso

2 Jun 2026

PMEN-D-26-00039

Representations of mental health, madness, mental illness and depression in the general population of French Guiana: similarities and differences between sociodemographic subgroups

PLOS Mental Health

Dear Dr. Nacher,

Thank you for submitting your manuscript to PLOS Mental Health. I am very sorry for the long delay, which was due to difficulties securing reviewers. After careful consideration of the reviewer reports, which we have now received, we feel that your paper has merit but does not fully meet PLOS Mental Health’s publication criteria as it currently stands. Therefore, we invite you to submit a revised version of the manuscript that addresses the points raised during the review process. You can find all of the comments below and in the attached document.

Please submit your revised manuscript by Jun 30 2026 11:59PM. If you will need more time than this to complete your revisions, please reply to this message or contact the journal office at mentalhealth@plos.org. When you're ready to submit your revision, log on to https://www.editorialmanager.com/pmen/ and select the 'Submissions Needing Revision' folder to locate your manuscript file.

Please include the following items when submitting your revised manuscript:

- A letter that responds to each point raised by the editor and reviewer(s). You should upload this letter as a separate file labeled 'Response to Reviewers'.
- A marked-up copy of your manuscript that highlights changes made to the original version. You should upload this as a separate file labeled 'Revised Manuscript with Track Changes'.
- An unmarked version of your revised paper without tracked changes. You should upload this as a separate file labeled 'Manuscript'.

Guidelines for resubmitting your figure files are available below the reviewer comments at the end of this letter.

As the corresponding author, your ORCID iD is verified in the submission system and will appear in the published article. PLOS supports the use of ORCID, and we encourage all coauthors to register for an ORCID iD and use it as well. Please encourage your coauthors to verify their ORCID iD within the submission system before final acceptance, as unverified ORCID iDs will not appear in the published article. *Only*  the individual author can complete the verification step; PLOS staff *cannot*  verify ORCID iDs on behalf of authors.

We look forward to receiving your revised manuscript.

Kind regards,

Karli Montague-Cardoso

Staff Editor

PLOS Mental Health

Journal Requirements:

1. Please include a complete copy of PLOS’ questionnaire on inclusivity in global research in your revised manuscript. Our policy for research in this area aims to improve transparency in the reporting of research performed outside of researchers’ own country or community. The policy applies to researchers who have travelled to a different country to conduct research, research with Indigenous populations or their lands, and research on cultural artefacts. The questionnaire can also be requested at the journal’s discretion for any other submissions, even if these conditions are not met.  Please find more information on the policy and a link to download a blank copy of the questionnaire here: https://journals.plos.org/mentalhealth/s/best-practices-in-research-reporting. Please upload a completed version of your questionnaire as Supporting Information when you resubmit your manuscript.

2. Please ensure that your Ethics Statement is available in its entirety at the beginning of your Methods section, under a subheading 'Ethics Statement'.

3. Please provide separate figure files in .tif or .eps format.

For more information about figure files please see our guidelines:  LINK

https://journals.plos.org/mentalhealth/s/figures

https://journals.plos.org/mentalhealth/s/figures#loc-file-requirements

4. We have noticed that you have uploaded Supporting Information files, but you have not included a list of legends. Please add a full list of legends for your Supporting Information files after the references list.

5. In the online submission form, you indicated that "For reasonable requests data may be made available by the CCOMS after approval from the commission nationale informatique et libertés.".

All PLOS journals now require all data underlying the findings described in their manuscript to be freely available to other researchers, either

1. In a public repository,

2. Within the manuscript itself, or

3. Uploaded as supplementary information.

This policy applies to all data except where public deposition would breach compliance with the protocol approved by your research ethics board. If your data cannot be made publicly available for ethical or legal reasons (e.g., public availability would compromise patient privacy), please explain your reasons by return email and your exemption request will be escalated to the editor for approval. Your exemption request will be handled independently and will not hold up the peer review process, but will need to be resolved should your manuscript be accepted for publication. One of the Editorial team will then be in touch if there are any issues.

If the reviewer comments include a recommendation to cite specific previously published works, please review and evaluate these publications to determine whether they are relevant and should be cited. There is no requirement to cite these works unless the editor has indicated otherwise.

Please review your reference list to ensure that it is complete and correct. If you have cited papers that have been retracted, please include the rationale for doing so in the manuscript text, or remove these references and replace them with relevant current references. Any changes to the reference list should be mentioned in the rebuttal letter that accompanies your revised manuscript. If you need to cite a retracted article, indicate the article’s retracted status in the References list and also include a citation and full reference for the retraction notice.

Additional Editor Comments (if provided):

[Note: HTML markup is below. Please do not edit.]

Reviewers' comments:

Reviewer's Responses to Questions

**Comments to the Author**

1. Does this manuscript meet PLOS Mental Health’s publication criteria?

Reviewer #1: Partly

Reviewer #2: Yes

**********

2. Has the statistical analysis been performed appropriately and rigorously?

Reviewer #1: No

Reviewer #2: Yes

**********

3. Have the authors made all data underlying the findings in their manuscript fully available (please refer to the Data Availability Statement at the start of the manuscript PDF file)?

The PLOS Data policy

Reviewer #1: Yes

Reviewer #2: Yes

**********

4. Is the manuscript presented in an intelligible fashion and written in standard English?

Reviewer #1: Yes

Reviewer #2: Yes

**********

Reviewer #1: It is really difficult to assess the relevance of the article, because the data is not cross-referenced. The paper studies a French territory resulting from colonization. At no time is there any mention of questions of social race and colonization. It is not possible to study a population of a colonized territory without the socio-demographic study mentioning the social race of the participants (« immigrant » or « first-generation immigrants » refer to nothing other than the status of arrival in a territory and does not allow us to establish either a social race or a social class, this is additional information). In French Guiana, there are indigenous, black, white people... The interviewees have religious, spiritual, and spatially situated material considerations that are not only related to « multiculturalism » but to relations of domination that should be questioned.

Similarly, the average age of the participants, their salary, their relationship to religion, and gender representation are to be compared with the general population: what does 1300 euros per month represent in French Guiana? Is religion present in general or only in your sample? etc. In short, it is difficult to distinguish social representations of madness in a nuanced way. Finally, there is a lack of links with the psychiatric institution in French Guiana: what is the importance of psychiatric institutions in this territory? Since when have there been asylums and hospitals?

Talking about madness and representations of madness without involving the psychiatric institution and its aura within the population is, in my opinion, damaging to the study. The imaginary of madness is built with the institution, otherwise the respondents would not talk about institutionalizing children perceived as « mad » for example.

Reviewer #2: Please see attachment. The main text is copied here:

Title: Representations of mental health, madness, mental illness and depression in the general population of French Guiana: similarities and differences between sociodemographic subgroups

1. MHPG (Mental Health in the General Population) Survey in French Guyana

This article explores social representations of madness, mental illness, and depression in the general population of French Guiana using data from the 2021 Mental Health in the General Population (MHGP/SMPG) survey. The study included 881 adults from the Cayenne area and examined how different sociodemographic groups perceive mental disorders, stigma, dangerousness, causes, and care pathways. The results show a clear distinction between depression, which is associated with sadness, suffering, and life events, and madness/mental illness, which remain linked to dangerousness, loss of control, violence, and social exclusion. Surprisingly, despite the strong cultural diversity of French Guiana, representations were globally homogeneous across subgroups. Socioeconomic factors (education and income) appeared more influential than cultural origin in shaping stigma and attitudes toward care. Professional healthcare was widely endorsed, whereas traditional or magico-religious explanations were infrequently declared. The study confirms the persistence of a hierarchical stigma structure, with madness remaining the most stigmatized category. Methodologically, the use of thematic coding and GEE multivariate models strengthens the robustness of the analyses. Overall, this article provides valuable epidemiological and sociocultural data on mental health representations in a highly multicultural territory and contributes meaningfully to anti-stigma and public mental health research.

2. Traditional, cultural, and religious care aspects

One of the interesting findings of this study concerns the apparent discrepancy between declared representations and actual practices regarding traditional and religious care. Respondents overwhelmingly reported a preference for professional medical and psychiatric care pathways when confronted with mental illness, depression, or madness. Religious, traditional, and magico-religious healers were only rarely mentioned as primary resources. This finding contrasts with common stereotypes frequently reported by healthcare professionals about the importance of traditional explanatory models in French Guiana. However, the authors appropriately discuss that social desirability bias may partly explain this result. Indeed, another study conducted on the same sample showed that people suffering from depression or post-traumatic stress disorder often resorted in practice to traditional healers and informal care systems. The article therefore highlights an important distinction between declared public discourse and real-life therapeutic itineraries. From an anthropological perspective, this reflects the coexistence of biomedical norms with persistent pluralistic systems of care deeply rooted in local cultures and migration histories. The discussion on these aspects is particularly relevant because it nuances simplistic cultural interpretations and underlines the role of socioeconomic vulnerability in shaping health-seeking behaviors.

3. Comparison with the MHPG survey in other French Overseas Territories

Given the limited number of studies on mental health in the general population (MHPG images and realities design) in the French overseas territories, it appears important to cite and compare results not only with New Caledonia but also with the two articles concerning the French Polynesia MHPG survey, the references of which are provided at the end of this analysis.

The findings from French Guiana may be certainly interestingly compared with previous MHPG investigations conducted in French Polynesia and other French overseas territories. In French Polynesia, and especially in studies involving Polynesian and Oceanian cultural contexts, representations of depression and mental illness often appeared more intertwined with collective, spiritual, and relational dimensions. In some Oceanian contexts, depression was not always clearly differentiated from madness and could be associated with explosive or violent behaviors, particularly in traditional Kanak representations previously described in New Caledonia. By contrast, in French Guiana, despite its exceptional multicultural composition, respondents showed relatively standardized representations close to those observed in mainland France and other French territories. Another important difference concerns traditional and religious explanatory models. In Polynesia, spiritual and family dimensions often occupy a more explicit place in narratives surrounding mental suffering and suicidal behavior, whereas in French Guiana such references were less openly declared in the survey. Nevertheless, both studies converge in demonstrating that social stigma remains strongly attached to “madness,” while depression is perceived as more understandable, treatable, and socially acceptable. Both surveys also confirm the major role of socioeconomic determinants and social inequalities in shaping access to care and representations of mental illness. Finally, the comparison highlights the great value of the MHPG methodology for cross-cultural psychiatric epidemiology within French overseas territories.

The comparison between French Guiana and French Polynesia becomes particularly interesting when examining traditional, cultural, and spiritual care practices. In the French Polynesian MHPG studies, although magico-religious explanations for mental illness were rarely explicitly declared in questionnaire responses (less than 0.5%), actual recourse to traditional care practices was much more frequent, reaching approximately 22–26% of respondents. This discrepancy between declared biomedical representations and real therapeutic itineraries is one of the major anthropological findings of the Polynesian studies.

In Polynesia, traditional healing practices remain deeply embedded in social and family life. Several complementary systems coexist with biomedical psychiatry. The tahua (traditional healer) may intervene in situations of psychological suffering, behavioral disorders, perceived spiritual imbalance, or suicidal crises. Traditional plant-based medicine (ra’au Tahiti) continues to occupy an important role, especially for anxiety, insomnia, somatic complaints associated with emotional distress, and culturally interpreted suffering. Likewise, traditional massages (taurumi) are not only physical treatments but may also carry symbolic, relational, and spiritual dimensions linked to restoration of harmony between body, mind, family, and environment.

The MHPG Polynesian studies showed that these practices are often integrated into pluralistic care pathways rather than being opposed to psychiatry. Patients and families may simultaneously consult psychiatrists, general practitioners, pastors, relatives, and traditional healers according to the perceived nature of the suffering. In the article on suicide risk and mental health in French Polynesia, recourse to traditional treatment was reported in approximately 26% of participants and was associated with specific sociocultural variables and suicidal vulnerability. The data further showed differences according to cultural identity, with stronger use among Polynesian and mixed-identity respondents than among Europeans or Chinese participants.

Compared with French Guiana, an important distinction emerges. In French Guiana, traditional and magico-religious practices appeared much less openly acknowledged in the declarative survey responses, despite evidence from clinical practice and complementary studies suggesting that such practices remain common. In Polynesia, by contrast, the existence of traditional healing systems seems more socially integrated and culturally legitimate, even when biomedical representations dominate public discourse. Another difference concerns the symbolic role of the family and collective identity in Polynesia. Traditional care often involves family participation, relational repair, spiritual explanations, and cultural continuity, especially in suicidal crises and depressive suffering.

These findings suggest that the apparent dominance of biomedical representations in both territories should not obscure the persistence of culturally embedded therapeutic systems. The Polynesian studies therefore contribute importantly to transcultural psychiatry by demonstrating how modern psychiatric care and indigenous healing traditions may coexist in complementary rather than antagonistic ways. This dimension is particularly valuable for understanding help-seeking behaviors, suicide prevention strategies, and culturally adapted mental health interventions in Pacific societies.

4. General conclusion on the interest and publishability of the article

This article is clearly of scientific and public health interest and appears suitable for publication. It addresses an original and insufficiently explored topic in a highly multicultural and socioeconomically vulnerable territory. The study benefits from a solid methodological framework combining qualitative thematic analysis with advanced multivariate statistical modeling. One of its major strengths lies in challenging preconceived assumptions regarding cultural heterogeneity in French Guiana by demonstrating the predominance of shared representations across groups. The discussion is balanced, nuanced, and appropriately acknowledges the study’s limitations. The article also contributes important reflections on stigma, social inequalities, help-seeking pathways, and the coexistence of biomedical and traditional representations. Beyond French Guiana, the findings have broader implications for transcultural psychiatry, psychiatric epidemiology, and anti-stigma strategies in multicultural societies. The manuscript is therefore both relevant and timely, and it constitutes a valuable contribution to the literature on mental health representations in overseas and culturally diverse populations.

References fo MHPG surveys in French Polynesia :

1. Amadéo S, Benradia I, David-Vanquin G, Rereao M, Meunier-Tuheaiva A, Favro P, Vilhem S, Fenni A, Nguyen NL, Goodfellow B, Sy A, Grand S, Jehel L, Roelandt JL. Représentations sociales et aspects anthropo-culturels de la santé mentale en Polynésie française dans l’enquête “Santé mentale en population générale : images et réalités”. L’Information Psychiatrique. 2022;98(7):530-544. doi:10.1684/ipe.2022.2465. (in FRENCH).

2. Amadéo S, Benradia I, Sy A, Rereao M, Favro P, David-Vanquin G, Meunier-Tuheaiva A, Lacoste J, Fenni A, Nguyen NL, Goodfellow B, Jehel L, Roelandt JL. Suicide risk and mental health in the general population of French Polynesia. Journal of International Medical Research. 2022;50(10):1-14. doi:10.1177/03000605221111273.

**********

what does this mean?). If published, this will include your full peer review and any attached files.

**Do you want your identity to be public for this peer review?**  If you choose “no”, your identity will remain anonymous but your review may still be made public.

For information about this choice, including consent withdrawal, please see our Privacy Policy

Reviewer #1: No

Reviewer #2: **Yes:** Stéphane Amadéo

**********

[NOTE: If reviewer comments were submitted as an attachment file, they will be attached to this email and accessible via the submission site. Please log into your account, locate the manuscript record, and check for the action link "View Attachments". If this link does not appear, there are no attachment files.]

# Author comment: Author response to Decision Letter 1

**DOI:** 10.1371/journal.pmen.0000655.r002

6 Jun 2026

Attachment Submitted filename: revision_letter_PMEN-D-26-00039.docx

# Peer review: Decision Letter 1

**DOI:** 10.1371/journal.pmen.0000655.r003

**Authors:** Karli Montague-Cardoso

24 Jun 2026

Representations of mental health, madness, mental illness and depression in the general population of French Guiana: similarities and differences between sociodemographic subgroups

PMEN-D-26-00039R1

Dear Pr. Nacher,

We are pleased to inform you that your manuscript 'Representations of mental health, madness, mental illness and depression in the general population of French Guiana: similarities and differences between sociodemographic subgroups' has been provisionally accepted for publication in PLOS Mental Health.

Before your manuscript can be formally accepted you will need to complete some formatting changes, which you will receive in a follow up email. A member of our team will be in touch with a set of requests.

Please note that your manuscript will not be scheduled for publication until you have made the required changes, so a swift response is appreciated.

IMPORTANT: The editorial review process is now complete. PLOS will only permit corrections to spelling, formatting or significant scientific errors from this point onwards. Requests for major changes, or any which affect the scientific understanding of your work, will cause delays to the publication date of your manuscript.

If your institution or institutions have a press office, please notify them about your upcoming paper to help maximize its impact. If they'll be preparing press materials, please inform our press team as soon as possible -- no later than 48 hours after receiving the formal acceptance. Your manuscript will remain under strict press embargo until 2 pm Eastern Time on the date of publication. For more information, please contact mentalhealth@plos.org.

Thank you again for supporting Open Access publishing; we are looking forward to publishing your work in PLOS Mental Health.

Best regards,

Karli Montague-Cardoso

Staff Editor

PLOS Mental Health

***********************************************************

Reviewer Comments (if any, and for reference):

Reviewer's Responses to Questions

**Comments to the Author**

Reviewer #1: All comments have been addressed

Reviewer #2: All comments have been addressed

**********

publication criteria?

Reviewer #1: Yes

Reviewer #2: Yes

**********

3. Has the statistical analysis been performed appropriately and rigorously?

Reviewer #1: Yes

Reviewer #2: Yes

**********

4. Have the authors made all data underlying the findings in their manuscript fully available (please refer to the Data Availability Statement at the start of the manuscript PDF file)?

The PLOS Data policy

Reviewer #1: Yes

Reviewer #2: Yes

**********

5. Is the manuscript presented in an intelligible fashion and written in standard English?

Reviewer #1: Yes

Reviewer #2: Yes

**********

Reviewer #1: The comments are adressed and answered. It cleared the misunderstanding.

Reviewer #2: This paper is now ready for publication

**********

what does this mean?). If published, this will include your full peer review and any attached files.

**Do you want your identity to be public for this peer review?**  If you choose “no”, your identity will remain anonymous but your review may still be made public.

For information about this choice, including consent withdrawal, please see our Privacy Policy

Reviewer #1: No

Reviewer #2: **Yes:** Pr Stephane AMADEO

**********
